# Mature Teratoma of the Uterus Removed with the IBS^®^ Integrated Bigatti Shaver: Case Report and Review of the Literature

**DOI:** 10.3390/jcm15072587

**Published:** 2026-03-28

**Authors:** Xiaoxiao Hu, Yanhua Zheng, Shanni Guo, Kaili Wang, Xia Yin

**Affiliations:** 1 Sino European Life Expert Centre (SELEC), Department of Gynecology and Obstetrics, Renji Hospital, Shanghai Jiao Tong University School of Medicine, Shanghai 200127, China; 2 Shanghai Key Laboratory of Gynecologic Oncology, Shanghai 200127, China

**Keywords:** uterine teratoma, mature teratoma, Integrated Bigatti Shaver, case report

## Abstract

**Background/Objectives**: Teratomas are the most common germ cell tumors, occurring mainly in the gonads. Extragonadal germ cell teratomas (EGGCTs) are less common but can develop anywhere along the midline structures, with uterine location being extremely rare. On an ultrasound, uterine teratomas are frequently misinterpreted as polyps or myomas. **Case Presentation**: We report a 27-year-old asymptomatic woman who was diagnosed with a mature uterine teratoma originating from the lower uterine segment and extending to the cervix. We treated this patient with the IBS^®^ Integrated Bigatti Shaver. This is the first reported case of the IBS^®^ being used to remove uterine teratomas. At follow-up, the patient recovered uneventfully and subsequently achieved a successful term pregnancy. **Conclusions**: Preoperative MRI is recommended for uterine teratomas. The IBS^®^ technique offers an effective and fertility-preserving approach for excising uterine teratomas, providing rapid procedure, superior visualization, and the prevention of tumor dissemination. In this report, we discuss the mechanism, diagnosis, and treatment of teratomas and review the previous literature.

## 1. Introduction

Teratomas are the most common tumors of germ cells, with an estimated incidence ranging from 1 in 20,000 to 1 in 40,000 live births [1]. Mature teratomas account for 97% of all teratomas, predominantly in the gonads such as the testes and ovaries. The annual incidence of mature cystic teratomas is estimated to range from 1.2 to 14.2 per 100,000 women [2]. Extragonadal germ cell teratomas (EGGCTs) are relatively rare and account for only 1–2% of all teratomas. They can occur anywhere in the midline structures, including the mediastinum, sacrum, retroperitoneum, and brain [3]. Uterine teratomas, as part of extragonadal tumors, are considerably rarer. 

Despite their extreme rarity, uterine teratomas present unresolved clinical issues. Preoperative diagnosis remains difficult, as they frequently mimic common intrauterine pathologies like submucosal fibroids or polyps. The choice of surgical approach is critically important, since the traditional electric resectoscope technique risks thermal damage to healthy endometrium and intraoperative tumor spillage, potentially increasing recurrence. These issues underscore the need for continued case reporting to refine diagnostic strategies, evaluate innovative techniques, and better understand tumor biology. 

Given the paucity of reported cases, we report a case of uterine teratoma in which the pioneering technique of the Integrated Bigatti Shaver (IBS^®^) was employed for excision.

## 2. Case Presentation

A 27-year-old asymptomatic woman, gravida 1 para 1 with a previous cesarean section, was referred to Renji Hospital Sino European Life Expert Centre in March 2023, four months after a mass was discovered during a routine transvaginal ultrasound examination.

**Figure 1:**
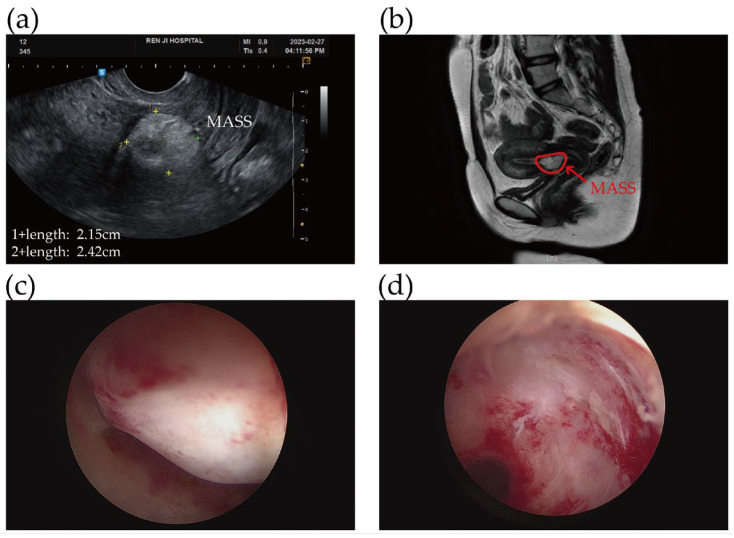
(**a**) Transvaginal ultrasound image showed that an isoechoic echo tumor measuring approximately 2.15 × 2.42 cm, originated from the lower uterine segment extending to cervix. (**b**) MRI study suggested an intracavitary lipid-based teratoma measuring 2.4 cm. (**c**) A myoma-like mass was seen in the lower uterine segment near the internal cervical os by hysteroscopy. (**d**) The tumor was completely enucleated after the IBS^®^ treatment.

Gynecologic examination findings were unremarkable. Transvaginal ultrasonography revealed a mass between the lower uterine segment and the cervix measuring 22 × 24 × 28 mm (Figure 1a). The adnexa and cervix appeared normal. Magnetic resonance imaging (MRI) findings were in favor of benign teratoma due to the presence of predominantly fatty signal and the absence of swollen lymph nodes (Figure 1b). Tumor markers including AFP, CA125, CA19-9 and CEA, were all within normal limits. Through the application of a hysteroscope, a 3 cm pedunculated, myoma-like lesion was discovered attached to the lower uterine segment close to the internal cervical os (Figure 1c). A surgical intervention was recommended. In March 2023, the mass was completely removed by the Integrated Bigatti Shaver without any complications in the day care unit (Figure 1d). During the IBS^®^ procedure, the tissue was simultaneously cut and aspirated, so there was no dispersal of the contents within the uterine cavity. All procedures were performed under intravenous anesthesia, similar to sedation protocols used in gastrointestinal endoscopy. The intraoperative morcellation time using the IBS system was about 3.5 min, while the total procedure duration was approximately 11 min.

**Figure 2:**
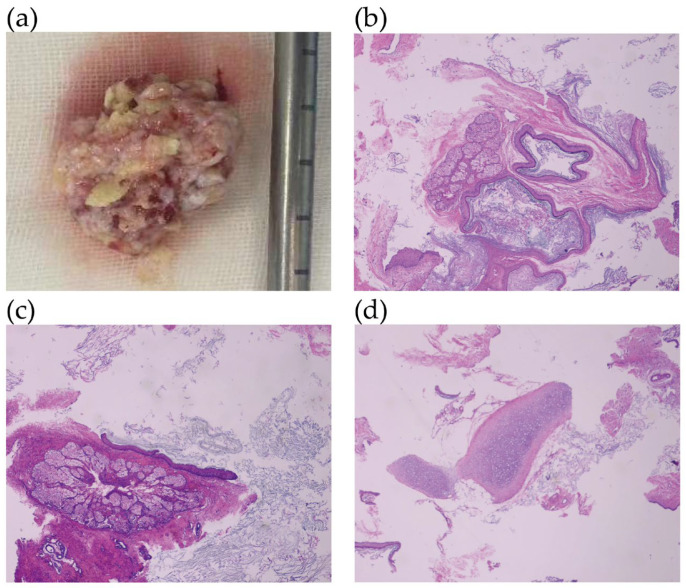
(**a**) Grossly, this mass was solid and contained white, lipoidal material, measuring 3.5 cm. (**b**–**d**) Histopathologic findings on H and E-stained Sections (40×) showed different types of tissues: (**b**) Skin keratinization; (**c**) sebaceous gland; (**d**) cartilage.

Grossly, this mass was solid and contained white, lipoidal material, measuring 3.5 cm (Figure 2a). Microscopically, a variety of tissues were visible: cartilage from the mesoderm, ectoderm tissue such as skin, sebaceous glands and squamous epithelium (Figure 2b–d). All the components were well differentiated and of adult type, meeting the criteria of a mature teratoma deriving from at least two embryonic layers. No immature neuroplastic tissue, which is the histologic evidence of malignancy, was observed in the vision.

**Figure 3:**
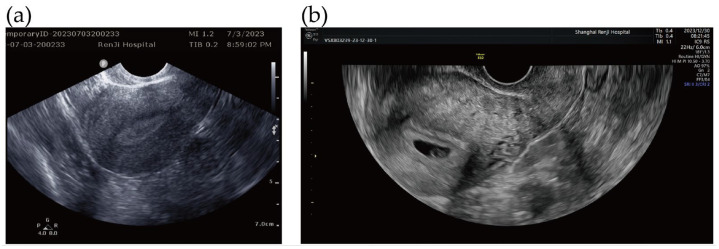
(**a**) Follow-up ultrasound obtained three months after hysteroscopic resection showed no residual mass and a well-healed uterine cavity. (**b**) Early pregnancy transvaginal ultrasound confirmed a viable intrauterine pregnancy after eight months of the surgery.

At the three-month postoperative follow-up, ultrasonography confirmed the absence of any residual mass (Figure 3a). She subsequently conceived, with an early ultrasound demonstrating an intrauterine pregnancy after 8 months of surgery (Figure 3b), and delivered a healthy full-term infant via cesarean section on 2 August 2024.

## 3. Discussion

EGGCTs are confusing and fascinating. They are histologically identical to germ cell tumors, but their biology is substantially different. Compared to the gonadal malignant teratomas (82.3%), the relative five-year survival rate of the extra-gonadal malignant teratomas (27.3%) is significantly lower (*p* < 0.01) [4]. 

Uterine teratomas, as a rare part of extra-gonadal tumors, differ from ovarian teratomas. A comprehensive review of reported uterine teratoma cases reveals 17 mature and 13 immature tumors, yielding an immaturity ratio of about 43.3% (13/30). However, given the extreme rarity of this neoplasm, this ratio should be interpreted with caution, as publication bias may overrepresent clinically aggressive immature teratomas in the literature. In contrast, the immaturity ratio for ovarian teratomas is well-documented to be less than 1% [5]. While it is generally believed that ovarian teratomas tend to affect young adults, uterine teratomas may influence somebody between the ages of 11 and 90 [6,7]. Furthermore, unlike ovarian teratomas for which ethnic epidemiological features have been described [8], data on ethnic predisposition for uterine teratomas are not available due to the extreme rarity of reported cases. A literature search identified 30 documented cases, none of which provided information on ethnic distribution.

The case of primary uterine teratoma was first reported in 1929 by Mann [9]. Since uterine teratomas are so fairly rare it is debatable whether they are primary neoplasms. A small number of primary uterine teratoma cases were subsequently documented, and several hypotheses on the origins of these tumors were proposed. One theory is that the tumor originated from residual fetal tissue that remained in the uterus for several decades after a ‘missed abortion’ or an ‘undelivered papyraceous twin’ [10]. A variety of heterotopic tissue can be observed in the uterus once secondary changes like organization or metaplasia take place. Currently, the most commonly recognized hypothesis is the parthenogenetic theory, which states that activated ovarian oocytes that had already finished the first meiotic division prior to ovulation are the source of uterine teratomas. This theory is supported by the finding that these teratomas have a normal 46,XX karyotype, with identical X chromosomes derived solely from the host [11]. In our case, the patient underwent cesarean surgery for dystocia eight years ago with no previous miscarriage history. Although restricted by the test conditions to identify the origin in our case, the second explanation seems to be more convincing. Regarding the potential association between the patient’s history of cesarean section and the occurrence of the uterine teratoma, a careful review of the literature reveals no documented evidence suggesting that cesarean section scars predispose patients to the development of uterine teratomas or other germ cell neoplasms.

Clinical presentations of uterine teratomas include abnormal uterine bleeding, pelvic pain, vaginal discharge, cervical polyps, and increased abdominal girth. Most tumor markers are within the normal range, while a few are slightly elevated. Uterine teratomas are challenging to preoperatively diagnose. Based on the available data, ultrasound typically reveals a moderately to highly echogenic mass with internal echogenic inhomogeneity which is often misdiagnosed as polyps and submucosal fibroids. This is attributed to the absence of classic hair and teeth inside the uterine teratomas, which is different from the features of the ovarian teratomas. MRI may offer more advantages in the differential diagnosis of uterine teratomas. Hysteroscopically, the lesion appeared as a pedunculated, myoma-like mass, consistent with previous reports that uterine teratomas are often indistinguishable from leiomyomas, polyps, or other pathology such as uterine tumors resembling ovarian sex-cord tumors (UTROSCTs) due to their solid consistency and surface appearance [12]. However, certain features may raise suspicion for teratoma, including a surface lacking visible vascularity, a uniform capsule, and a consistency softer than that of a typical myoma yet notably firmer than an endometrial polyp, with an absence of overlying endometrium, but these findings are not definitive. The pathology, however, is still the gold standard for diagnosis. 

The main treatment for uterine teratomas is surgery. The choice of the best treatment method depends on the site of the tumor, age, size, and pathologic typing. Hysteroscopic or laparoscopic surgery is the most common surgical approach for uterine teratomas, with hysteroscopy being the preferred method for lesions located within the uterine cavity.

As far as we know, the electric resectoscope is widely used and remains the instrument of first choice for the excision of the pathology in the cavity. Unfortunately, due to technical difficulties such as a limited visual field and prolonged operative time, fragmented tissue tends to remain in the uterine cavity, requiring the repeated insertion and removal of instruments to clear the debris and restore a clear surgical view. More importantly, it is widely recognized that the intraoperative spillage of teratoma contents significantly increases the risk of postoperative recurrence or metastasis. If the postoperative pathology reveals malignancy, the consequences could be even more severe. 

Mechanical hysteroscopic tissue removal (mHTR) systems offer a potential solution to these challenges. Among currently available hysteroscopic tissue removal systems, the IBS^®^ system offers the unique advantage of simultaneous cutting and aspiration, with adjustable suction power and rotation speed that can be tailored intraoperatively to tissue consistency, allowing the effective resection of both soft and hard components, as encountered in uterine teratomas. However, while the majority of operative hysteroscopic procedures, such as polypectomies and myomectomies, can be successfully performed with the IBS^®^, its application in the management of uterine teratomas has not been previously documented. The key feature of the IBS^®^ proved particularly valuable in our case, where the tumor originated from the lower uterine segment and extended into the cervical canal, a narrow region where maintaining a clear operative field is challenging. Despite these challenges, the IBS^®^ successfully removed the uterine teratoma in a single 3.5 min procedure, without complications and with complete preservation of the endometrium against thermal damage. By continuously removing tissue debris during resection, the IBS^®^ provided enhanced visualization compared to TruClear or MyoSure, which may require more frequent fluid management or instrument exchange to clear the cavity. Furthermore, IBS’s integrated suction mechanism may help minimize the risk of tumor cell dissemination by promptly removing resected fragments. 

According to the previous literature (Table 1), excision of the lesion is sufficient for mature uterine teratomas. Although benign teratoma in one case underwent malignant transformation, there has been no evidence of recurrence. The role of adjuvant chemotherapy in malignant uterine teratomas is not yet clear. In ovarian germ cell tumors, the risk of recurrence ranges from 25% to almost 100%, so three courses of bleomycin, etoposide, and cisplatin (BEP) are recommended in completely resected patients to prevent recurrence [13]. It is possible that the therapeutic effect of BEP is also suitable for malignant uterine tumors.

This case report was prepared in accordance with the CARE guidelines, and the completed CARE checklist is provided in the Supplementary Materials.

## 4. Conclusions

The case we reported demonstrates the successful application of hysteroscopic resection using the IBS^®^ for a uterine mature teratoma, which resulted in the preservation of fertility and the subsequent achievement of a term pregnancy. The favorable reproductive outcome underscores how this procedure effectively manages these rare uterine pathologies, as it prevents tumor dissemination and simultaneously meets the fertility aspirations of young patients.

## Figures and Tables

**Table 1:** Clinical and pathological characteristics of patients with primary uterine mature teratomas in English literature.

Author	Year	Age	Symptoms	Site of Tumor	Histology	Treatment	Follow-Up	Treatment of Relapse and Prognosis
Mold J.W. [14]	1969	34	Vaginal bleeding	Uterine cavity	Mature teratoma	TH-BSO	No relapse after 3 years	
Hanai J. [15]	1977	26	Uterine cervix mass	Uterine cervix	Mature teratoma	tumor excision	Not specified	
Martin E. [11]	1979	43	Pelvic pain	Uterine cavity	Benign teratoma	TH-BSO	Not specified	
Iwanaga S. [16]	1990	28	Amenorrhea	Uterine cervix	Mature teratoma	tumor excision	No relapse after 3 years	
Cappello F. [17]	2000	55	Asymptomatic	Uterine corpus	Mature teratoma with thyroid differentiation	TH	Not specified	
Lim S.C. [18]	2003	27	Leukorrhea and vaginal spotting	Uterine cervix	Mature teratoma with exuberant lymphoid elements	tumor excision	Not specified	
Sissons M.C. [19]	2003	51	Dysmenorrhea and vaginal discharge	Uterine cavity	Benign teratoma	tumor excision	No relapse after 4 years	
Papadia A. [20]	2007	58	Asymptomatic	Uterine cavity	Mature teratoma	HR	Not specified	
Newsom-Davis T. [7]	2009	82	Postmenopausal bleeding	Uterine corpus	Benign teratoma/immature teratoma	HR TH-BSO	Benign teratoma/immature teratoma	(ET+TP) × 3 + lymph node dissection, then die after one month
Wang W.C. [21]	2011	46	Abdominal mass	Uterine corpus	Mature teratoma	Transabdominal tumor excision	No relapse after 6 months	
Kamgobe E. [22]	2016	21	Abdominal pain and distension, fever, and abnormal vaginal discharge	Uterine corpus	Mature teratoma	TH	No relapse after 8 months	
Galko J. [23]	2017	37	Vaginal bleeding	Uterine corpus	Mature teratoma	HR	No relapse after 15 months	
Loo Z.X. [24]	2019	26	Abdominal pain, irregular menstruation, and hypomenorrhea	Anterior uterine serosal layer	Mature teratoma	Transabdominal tumor excision	Not specified	
Lin T.C., Chen T.H. [25]	2020	37	Abdominal pain	Uterine corpus	Mature teratoma	Transabdominal tumor excision	Not specified	
Rouholamin S. [26]	2023	35	Abnormal uterine bleeding	Uterine cavity	Mature teratoma	Transabdominal tumor excision	Not specified	
Li M. [27]	2024	40	Uterine cervical mass	Uterine cervix	Mature teratoma	tumor excision	No relapse after 3 months	
AlAshqar A. [28]	2024	44	Vaginal bleeding	Uterine cavity	Mature teratoma	TH-BS	Not specified	

Abbreviations: HR, hysteroscopic resection; TH, total hysterectomy; TH-BS, total hysterectomy with bilateral salpingectomy; TH-BSO, total hysterectomy with bilateral salpingo-oophorectomy; Chemotherapy regimens: ET, etoposide plus paclitaxel; TP, paclitaxel plus cisplatin.

## Data Availability

The original contributions presented in the study are included in the article, further inquiries can be directed to the corresponding author.

## Supplementary Materials

The following supporting information can be downloaded at: https://www.mdpi.com/article/10.3390/jcm15072587/s1, CARE Checklist.

## Abbreviations

The following abbreviations are used in this manuscript:

IBS	Integrated Bigatti Shaver
EGGCTs	Extragonadal Germ Cell Teratomas
MRI	Magnetic Resonance Imaging
UTROSCTs	Uterine Tumors Resembling Ovarian Sex-Cord Tumors
mHTR	mechanical Hysteroscopic Tissue Removal
BEP	Bleomycin, Etoposide, Cisplatin
TH-BSO	Total Hysterectomy with Bilateral Salpingo-oophorectomy
TH-BS	Total Hysterectomy with Bilateral Salpingectomy
TH	Total Hysterectomy
HR	Hysteroscopic Resection
ET	Etoposide, Paclitaxel
TP	Paclitaxel, Cisplatin
