# Supplementary material for: Mature Teratoma of the Uterus Removed with the IBS® Integrated Bigatti Shaver: Case Report and Review of the Literature
Source: J Clin Med. 2026 Mar 28;15(7):2587. doi: 10.3390/jcm15072587 (PMC13073115; doi:10.3390/jcm15072587)
Supplement: Supplementary file 1 [file jcm-15-02587-s001.zip › jcm-4143948-supplementary.pdf]

CARE
case report guidelines

CARE Checklist of information to include when writing a case report

(cc) BY-NC-ND

Topic	Item	Checklist item description	Reported on Line
**Title**	1	The diagnosis or intervention of primary focus followed by the words "case report"	Line 2-4
**Key Words**	2	2 to 5 key words that identify diagnoses or interventions in this case report, including "case report"	Line 26
**Abstract (no references)**	3a	Introduction: What is unique about this case and what does it add to the scientific literature?	Line 12-21
	3b	Main symptoms and/or important clinical findings	Line 16-21
	3c	The main diagnoses, therapeutic interventions, and outcomes	Line 16-21
	3d	Conclusion—What is the main "take-away" lesson(s) from this case?	Line 21-25
**Introduction**	4	One or two paragraphs summarizing why this case is unique (**may include references**)	Line 29-44
**Patient Information**	5a	De-identified patient specific information	Line 48-51
	5b	Primary concerns and symptoms of the patient	Line 48-51
	5c	Medical, family, and psycho-social history including relevant genetic information	Line 48-51;121-123
	5d	Relevant past interventions with outcomes	/
**Clinical Findings**	6	Describe significant physical examination (PE) and important clinical findings	Line 52-59
**Timeline**	7	Historical and current information from this episode of care organized as a timeline	Line 48-91
**Diagnostic Assessment**	8a	Diagnostic testing (such as PE, laboratory testing, imaging, surveys).	Line 52-83
	8b	Diagnostic challenges (such as access to testing, financial, or cultural)	Line 129-145
	8c	Diagnosis (including other diagnoses considered)	Line 73-83
	8d	Prognosis (such as staging in oncology) where applicable	Line 84-91
**Therapeutic Intervention**	9a	Types of therapeutic intervention (such as pharmacologic, surgical, preventive, self-care)	Line 59-72
	9b	Administration of therapeutic intervention (such as dosage, strength, duration)	Line 62-66
	9c	Changes in therapeutic intervention (with rationale)	/
**Follow-up and Outcomes**	10a	Clinician and patient-assessed outcomes (if available)	Line 84-91
	10b	Important follow-up diagnostic and other test results	Line 84-91
	10c	Intervention adherence and tolerability (How was this assessed?)	Line 84-91;193-198
	10d	Adverse and unanticipated events	/
**Discussion**	11a	A scientific discussion of the strengths AND limitations associated with this case report	Line 146-175
	11b	Discussion of the relevant medical literature **with references**	Line 176-191
	11c	The scientific rationale for any conclusions (including assessment of possible causes)	Line 146-175
	11d	The primary "take-away" lessons of this case report (without references) in a one paragraph conclusion	Line 193-198
**Patient Perspective**	12	The patient should share their perspective in one to two paragraphs on the treatment(s) they received	/
**Informed Consent**	13	Did the patient give informed consent? Please provide if requested	Yes ☑ No ☐
